# Blackcurrant Pomace Extract as a Natural Antioxidant in Vienna Sausages Reformulated by Replacement of Pork Backfat with Emulsion Gels Based on High Oleic Sunflower and Flaxseed Oils

**DOI:** 10.3390/gels10080534

**Published:** 2024-08-13

**Authors:** Nicoleta Cîrstea (Lazăr), Violeta Nour, Alexandru Radu Corbu, Georgiana Gabriela Codină

**Affiliations:** 1 Faculty of Food Science and Engineering, Dunărea de Jos University of Galati, Domnească Street 111, 800201 Galati, Romania; nl135@student.ugal.ro; 2 Department of Horticulture & Food Science, University of Craiova, 13 AI Cuza Street, 200585 Craiova, Romania; corbu_lx@yahoo.co.uk; 3 Faculty of Food Engineering, Stefan cel Mare University of Suceava, 720229 Suceava, Romania; codina@fia.usv.ro

**Keywords:** meat product, reformulation, blackcurrant pomace, oxidative stability, fatty acid profile, technological properties, color, texture, sensory properties

## Abstract

The incorporation of a blackcurrant pomace extract (BPE) at 2.5%, 5.0% and 10.0% into an emulsion gel based on high oleic sunflower and linseed oils was examined in order to obtain a functional ingredient to be used as a pork backfat replacer in Vienna sausages. The replacement of the pork backfat with the control emulsion gel reduced the cooking loss but negatively affected the color by decreasing L* and a* values as compared with the traditional product. A decrease in the n-6/n-3 ratio from 10.99 to around 1.54 (by 7 times) was achieved through reformulation, while the PUFA/SFA ratio increased from 0.49 to 1.09. The incorporation of BPE did not have a major impact on the fatty acid profile and improved color by increasing redness, but negatively affected the texture by increasing hardness, gumminess and share force as compared with the sausages reformulated without extract. BPE reduced the pH and the thermal stability of the emulsion gels, increased cooking loss and decreased moisture retention in sausages. BPE increased the oxidative stability of Vienna sausages enriched in polyunsaturated fatty acids; however, the incorporation of BPE into the emulsion gels above 5% affected the sensory scores for appearance, texture and general acceptability of the reformulated sausages.

## 1. Introduction

Vienna sausage is a ready-to-eat thin sausage traditionally made of finely ground pork and beef meats stuffed in a casing of sheep’s intestine, cooked and then smoked at a low temperature [1]. Although they are one of the most widely consumed emulsified meat products, Vienna sausages are not typically regarded as a healthy food due to their high content of salt and preservatives, as well as their high animal fat content (20–30%) dominated by saturated fats. Nowadays, it is well known that a high consumption of saturated fatty acids and a high dietary n-6/n-3 ratio are associated with inflammatory processes, lipotoxicity, high serum cholesterol and low-density lipoproteins (LDL) levels, which are risk factors for the onset and severity of certain chronic diseases such as cardiovascular disease, cancer, diabetes, and neurodegenerative diseases [2,3,4]. The number of people who are aware of the necessity of having a healthy diet has continuously increased [5], together with their demand for meat products with low fat content, healthier lipid profiles, and functional components [6]. A way to meet these requirements is the reformulation of meat products by lowering the fat content and/or by replacing the added pork backfat with oils rich in unsaturated fatty acids, preferably in n-3 fatty acids [7,8].

However, the direct substitution of animal fat with vegetable oils is a major challenge since animal fat is essential in the formation of stable meat emulsions and makes a major contribution to the technological, textural, and sensory properties of emulsified meat products [3,9,10,11]. To overcome these challenges, meat researchers have investigated the entrapment of the oils in solid oil-structured emulsions, such as emulsion gels and oleogels, possessing semi-solid textural properties similar to animal fats, and able to retain water and fat in the meat product and to protect the incorporated lipids from oxidation [12,13]. These structures are formulated by the incorporation of a cold gelling agent based on proteins, polysaccharides or their combinations into a protein-stabilized emulsion containing the oil [14,15,16]. Several studies have been conducted on the reformulation of emulsified meat products such as Bologna sausages [3,7,17] and frankfurters [18,19,20] by replacing pork backfat with emulsion gels containing a mixture of healthy oils. Recently, our research team reformulated Bologna sausage by totally replacing pork backfat with an emulsion gel containing olive, walnut, and chia oils, stabilized with soy protein isolate, transglutaminase, and chitosan [21]. The reformulation was effective in improving the lipid profile and it did not significantly affect the technological properties and the overall acceptance of the product by the consumers. However, a decrease in oxidative stability was noticed in the reformulated product as compared with the control.

A wide range of edible oils have been used to formulate emulsion gels, alone or in combinations, including sunflower, olive, sesame, chia, flaxseed, coconut, walnut and algal oils [6,13,22]. High oleic sunflower oil has become increasingly popular due to its high content of oleic acid (up to 65%), mild flavor and affordable price. Da Silva et al. [3] used this oil to produce an oleogel rich in oleic acid used as a pork backfat replacer in Bologna-type sausages, while it was previously incorporated into oleogels intended to replace shortenings in bakery products [23,24]. Linseed oil also proved to be a good candidate for incorporation into emulsion gels to increase the n-3 polyunsaturated fatty acids (n-3 PUFAs) content of the meat products due to its high content of α-linolenic acid (~55%) [19,25]. However, the high unsaturation degree of the oils makes them more susceptible to lipid oxidation as compared with animal fats; therefore, the reformulated meat products require additional strategies to enhance their oxidative stability [4,21]. Oxidation is one of the main factors in the quality deterioration and reduction in shelf life of meat products [26] by causing discoloration, the formation of the characteristic rancid flavor and undesirable changes in texture [27]. In addition to lipid oxidation, which is accompanied by losses of essential fatty acids and vitamins, protein oxidation occurs and determines the loss of amino acids and alterations in their functionality [28]. Moreover, oxidation results in the formation of harmful toxic compounds, which could increase the risk of chronic diseases such as atherosclerosis and cancer through the induction of inflammation [29].

To slow down oxidation reactions, synthetic antioxidants, such as butylated hydroxyl toluene (BHT), butylated hydroxyl anisole (BHA) or tert-butylhydroquinone (TBHQ), could be added to meat products [30]. However, consumers regard with suspicion the use of chemical additives as they are associated with toxicological and carcinogenic effects [29,31]. The increasing demand of consumers for healthier meat products has promoted the intensification of studies on the use of plant extracts or plant-derived compounds as alternatives for synthetic antioxidants in meat products [28,32]. Fruits, spices, herbs and essential oils, containing high levels of phenolic compounds and other antioxidants, have been tested as potential sources of antioxidant compounds to be used in meat products [12,22,26,27,33,34,35].

Nowadays, berries are widely consumed because they represent an important source of bioactive compounds and antioxidants [36] with immense health benefits and medicinal properties [37]. A series of studies on the use of berry extracts as natural antioxidants for the inhibition of lipid and protein oxidation in meat and meat products have been developed [4,38,39], but just a few of them used the by-products from fruit processing for this purpose [9,40,41]. Both wild and cultivated blackcurrants (*Ribes nigrum* L.) represent a rich source of polyphenols, especially phenolic acids, anthocyanins, flavonols, condensed tannins, and hydrolyzable tannins [42]. Because of their astringency, blackcurrants are mainly processed into juices, jams, jellies, and alcoholic beverages [43]. After the processing of blackcurrants into juice, a high amount of pomace is generated, comprising skins, pulp, and seeds. Blackcurrant pomace is extremely valuable due to its abundance in fiber, fruit acids, anthocyanins and other phenolic compounds [36,44]; therefore, the recovery and use of this by-product to produce functional food ingredients has gained increased interest.

This study aimed to evaluate the feasibility of Vienna sausage reformulation by totally replacing pork backfat with an emulsion gel containing high oleic sunflower and flaxseed oils, as well as blackcurrant pomace extract, as a natural antioxidant. The nutritional, physicochemical, technological, textural, and sensory properties of reformulated Vienna sausages were compared with control samples made with pork backfat, and with samples reformulated with emulsion gel without blackcurrant pomace extract.

## 2. Results and Discussion

### 2.1. Total Phenolic Content, Total Anthocyanins Content and Antioxidant Capacity of Dried Blackcurrant Pomace and Blackcurrant Pomace Extract

Dried blackcurrant pomace was analyzed for TPC, TAC and RSA using spectrophotometric methods. A total phenolic content of 26.2 mg GAE/g was found in dried blackcurrant pomace, in good agreement with previous studies reporting 24.34 mg GAE/g [36] or 37.5 mg GAE/g [45] in the dehydrated blackcurrant by-product of juice production. After extraction in water at 80 °C, a total phenolic content of 590 mg GAE/L was found in blackcurrant pomace extract (BPE). Blejan et al. [46] found a TPC value between 500 mg GAE/L for BPE made in water and 1618 mg GAE/L for BPE made in 60% ethanol by ultrasonic assisted extraction. Phenolic acids, anthocyanins, flavonols, condensed tannins, and hydrolyzable tannins are the most important bioactive compounds in blackcurrant pomace [42]. A total anthocyanins content of 3.68 mg CGE/g was found in the dried blackcurrant pomace via the pH differential method. Previously, Sójka and Król [47] reported 3.44–10.46 mg/g, while Gagneten et al. [45] found 18.0 mg cyanidin-3-glucoside/g in the dehydrated blackcurrant pomace. An anthocyanin content of 18.67 mg CGE/L was found in the blackcurrant pomace extract. Blejan et al. [46] found that ultrasonic-assisted extraction (UAE) was more effective than maceration in extracting anthocyanins from dried blackcurrant pomace, and reported between 12.61 and 24.19 mg CGE/L in the blackcurrant pomace extract made in water and 60% aqueous ethanol, respectively.

As for the DPPH radical scavenging activity assay, a value of 25.5 μmol Trolox/g was found in the dried blackcurrant pomace, and 0.72 mmol Trolox/L in the extract. The results regarding antioxidant activity derived in previous studies vary widely depending on the solvent and extraction conditions. Blejan et al. [48] reported an antioxidant activity in the range 0.52–1.01 mmol Trolox/L in blackcurrant pomace extracts obtained through UAE in different solvents. The blackcurrant pomace extracts made in 60% ethanol exhibited the highest radical-scavenging activity (1.63 mmol Trolox/L) [46]. These data point out the high antioxidant potential of the blackcurrant pomace and, together with its color properties, justify the attempt to incorporate it into meat products requiring antioxidant protection.

### 2.2. Color, pH and Stability of Emulsion Gels

EG0, EG2.5, EG5.0, and EG10.0 were produced by replacing 0%, 2.5%, 5.0% and 10.0% of the water used in gel formulation with blackcurrant pomace extract, respectively. Homogeneous, smooth, and stable emulsion gels were obtained from all formulations. The colors of the emulsion gels are important, considering their influence on the color of the reformulated meat product. The control emulsion gels (EG0) had a yellowish-cream color, determined mainly by the color of the soy protein isolate and of the oil mixture. Although the blackcurrant pomace extract had a light rose color, which seemed to positively affect the color of the meat product, the addition of the extract caused reductions in the L*, a* and b* values. This behavior can be attributed to the degradation and color changes of the anthocyanins from BPE, which turn blue and green at pH values higher than 7, as found in emulsion gels [49]. The L* values decreased more as the level of extract addition increased. Moreover, in the emulsion gels with higher extract incorporation levels (EG5.0 and EG10.0), the L* values decreased significantly during storage (Table 1).

The pH of the control emulsion gel (pH = 8.36, EG0) was higher than the pH values reported previously [18] in emulsion gels made with other stabilizing agents, mainly as a result of chitosan incorporation [25]. The higher pH values contributed to the good phase separation stability of the emulsion gels (total fluid release of 3.24% after 14 days of storage). The total fluid release significantly increased as a result of the BPE addition, indicating a decrease in the emulsion gel’s stability. This increase was greater with higher levels of extract incorporation, and it can be attributed to the significant decrease in pH (*p* < 0.05) that the BPE addition generated (Table 1). As expected, pH increased during storage in all formulations as a result of protein breakdown by endogenous or microbial enzymes, resulting in the production of amines, ammonia and other degradation products [39,50].

### 2.3. Texture Properties of Emulsion Gels

The values of the emulsion gels’ texture parameters at 0, 7 and 14 days of storage at 4 °C are shown in Table 2. The results show that the incorporation of BPE into the emulsion gel resulted in a statistically significant (*p* < 0.05) decrease in hardness, cohesiveness, adhesiveness and resilience, while springiness was not significantly (*p* > 0.05) affected. The adhesiveness decreased with increases in the extract incorporation up to almost three times, while the resilience decreased by up to 15.8% (EG10.0) as compared with the control (EG0).

These modifications of the textural properties may be attributed to the decrease in alkalinity in the emulsion gels as a result of BPE addition. Previous studies have shown that the structural changes and gel textures of soy-isolated proteins are favored by increasing pH under alkaline conditions. Sun et al. [51] found that the alkaline pH-shifting treatment increased the surface hydrophobicity of SPI, with a significant growth observed between pH 7.0 to 8.0 caused by the structural change in SPI at an alkaline pH. In addition, the solubility of SPI increased with the increasing alkalinity due to the greater expansion of the protein’s structure, exposing more hydrophobic amino acids. Consequently, they reported an increase in SPI gel hardness with increasing alkalinity due to the SPI’s conformational unfolding, which exposes more active sites favorable for transglutaminase cross-linking.

Hardness, adhesiveness, gumminess and chewiness significantly increased in all samples during 7 days of storage (4 °C), while springiness and resilience decreased. After 7 days of storage, the emulsion gels made with BPE were harder and more gummy than the controls. These variations may be attributed to the increase in the pH values during storage, but mostly to the greater degree of fluid release in emulsion gels with BPE incorporation, as mentioned earlier.

### 2.4. Proximate Composition and Energy Values of Vienna Sausages

The sausages of the formulations VSEG0, VSEG0.5, VSEG1.0, and VSEG2.0 were manufactured following the same recipe and technology as control Vienna sausages (VSC), except that pork backfat was replaced with EG0, EG2.5, EG5.0, and EG10.0 emulsion gels, respectively. The proximate composition and energy values of control and reformulated Vienna sausages are shown in Table 3. For control sausages, the values for moisture, fat, protein and ash were 58.57%, 20.32%, 12.66% and 2.26%, respectively. Kang et al. [52] found 56.44% moisture, 22.91% fat and 15.21% protein content in control chicken breast Vienna sausages, while Berasategi et al. [22] reported 14% protein content in the traditional Bologna sausage. In our study, the reformulation of Vienna sausages led to a decrease in the fat content between 6.54% and 13.79%. The greatest decrease in the fat content was observed in reformulated samples without the addition of by-product extract (VSEG0). The reformulated Vienna sausages had 4.55 to 9.56% lower energy values than the controls, mainly due to their lower fat content. Both protein and ash contents increased in all reformulated sausages, but the increases were not significant (*p* > 0.05). A slight tendency towards an increased ash content was noticed in reformulated samples, which was previously reported in reformulated frankfurters [53] or dry fermented sausages [54], and it was attributed to the higher ash content of the emulsion gel derived from the protein isolate as compared with the pork backfat.

The different levels of BPE addition in sausages did not significantly influence the contents of moisture, fat, protein and ash. Agregán et al. [28] found no significant changes regarding the proximate composition after the incorporation of different concentrations of *Fucus vesiculosus* extracts in pork patties. Kang et al. [52] reported slight but significant decreases in the fat content in Vienna sausages reformulated via the partial replacement of meat with emulsion manufactured with soybeans, while Heck et al. [12] also found that lipid reformulation reduced the fat content without affecting the protein and ash contents of raw and cooked burgers (*p* > 0.05).

### 2.5. Fatty Acid Profile

The fatty acid composition and nutritional indices of control and reformulated Vienna sausages are presented in Table 4. The fatty acid profile was dominated by MUFA in both control and reformulated sausages. However, the MUFA content increased from 46.78% in controls to an average percentage of 51% in reformulated sausages due to the use of the high oleic sunflower oil in the emulsion gel formulation (75% of the oil mixture). The content of PUFA increased as a result of reformulation from 17.39% to around 25%, while SFA decreased considerably (from 35.49% to around 23.3%).

Oleic acid was the most abundant fatty acid in both the control (43.25%) and reformulated (49.15–49.51%) sausages. However, control sausages contained substantial amounts of palmitic (21.87%) and stearic acids (11.2%), as well as a considerable amount of linoleic acid (14.76%). Important reductions in myristic (41.8%), palmitic (35.3%) and stearic (28.8%) acids were found as a result of reformulation, which is beneficial to human health as these saturated fatty acids are positively associated with increased triglycerides and low-density lipoprotein (LDL) cholesterol [4].

The content of linoleic acid (C18:2n-6) was found within the same limits in reformulated sausages as in controls, while the content of α-linolenic acid (C18:3n-3) increased by around ten times (from 0.93% in controls to around 9.33% in reformulated sausages). These variations in fatty acid profile may be attributed to the replacement of pork backfat with the emulsion gel containing 25% linseed oil, which is well known to be rich in α-linolenic acid (50–55% content). Similar results have been reported in previous studies as a result of pork backfat replacement in meat products with emulsion gels based on vegetable oils rich in mono- and polyunsaturated fatty acids [28,55]. PUFA/SFA increased from 0.49 in control sausages to 1.09 in reformulated ones, which greatly exceeds the threshold of 0.49 above which CVD risk reduction has been previously reported [56].

The reformulated Vienna sausages could be claimed as “high unsaturated fat” according to Regulation (EC) No 1924/2006 and Commission Regulation No 432/2012, since at least 70% of the fatty acids (76.36–76.74%) come from unsaturated fat. The n-6/n-3 ratio strongly decreased from 10.99 to around 1.54 (by more than 7 times) in the reformulated sausages as compared with the controls, while the PUFA/SFA ratio increased from 0.49 to 1.08. The n-6/n-3 ratio of reformulated Vienna sausages (1.51–1.57) meets the nutritional guidelines, which suggest that the target n-6/n-3 ratio should be balanced between 1:1 and 2:1 in a healthy diet [57], as it was demonstrated that the consumption of diets with high n-6/n-3 ratios promotes inflammatory reactions and chronic diseases, including cardiovascular and neurodegenerative diseases, and certain types of cancer.

Based on their thrombogenic potential, i.e., the tendency to form clots in blood vessels, fatty acids were divided into pro-thrombogenic (C12:0, C14:0, and C16:0) and anti-thrombogenic fatty acids (MUFA, n-3 and n-6 PUFA). The atherogenic (AI) and thrombogenic (TI) indexes as well as the h/H ratio are healthiness parameters that characterize the effects of dietary fats in the prevention of cardiovascular diseases, inflammations and chronic diseases. The reformulation of Vienna sausages contributed to the decrease in both indexes, from 0.45 to 0.23 for the AI and from 0.97 to 0.36 for the TI, and to the almost doubling of the h/H index (from 2.52 to 4.83–4.98), demonstrating an atherogenic and thrombogenic potential of the reformulated products about two times lower as compared with the controls. Many previous studies reported major reductions in the n-6/n-3 ratio, and in the thrombogenic and atherogenic indices, as a result of meat product reformulation [6,12,19,58,59]. As similarly reported by Berasategi et al. [22] in Bologna-type sausages stabilized with an extract of *Melissa officinalis*, no significant effect of the antioxidant extracts’ addition was noticed over the PUFA n-6 and PUFA n-3 contents. However, the reformulated sausages with antioxidant extracts showed higher contents of octadecatetraenoic (C18:4n-3) and eicosadienoic (C20:2n-6) acids as compared with those reformulated without antioxidant extracts (VSEG0). Similar very slight but significant differences have been reported previously, and they have been attributed to the capacity of the natural extracts to delay the degradation of unsaturated fatty acids [60].

### 2.6. Color and pH of Vienna Sausages

Table 5 shows the results for the CIEL*a*b* color parameters and pH of control and reformulated Vienna sausages at 0, 7, 14 and 21 days of refrigerated storage (4 °C).

After processing, the replacement of pork backfat with the control emulsion gel determined a significant (*p* < 0.05) increase in L* values and a decrease in redness (a* values), while yellowness (b* values) did not undergo significant changes. The incorporation of BPE into emulsion gels determined a progressive decrease in L* values with increasing levels of addition and an increment in the a* and b* values in the reformulated Vienna sausages. The increase in the a* values, considered favorable to the color of the meat product, may be attributed to the slightly acidic pH of the sausage paste (6.1–6.5), which causes the color of the anthocyanins in the extract to remain red, unlike in the emulsion gels, whose pH is in the basic range (7.0–8.0), where anthocyanins turn blue or green. The higher redness in the reformulated sausages manufactured with BPE can be explained also via the protection of the red color by the antioxidants from the natural extract. The stabilizing effects of different natural antioxidants on redness (a* values) have been reported in previous studies using avocado by-products [61], algae [28], oregano [62] or grape seed [63] extracts. A progressive loss of lightness was noted, while redness (a*) did not seem to follow any specific trend over storage time.

The pH values significantly (*p* < 0.05) increased as a result of reformulation with the control emulsion gel (EG0) due to its high pH value (8.36 ± 0.03, Table 1). The pH values decreased progressively in the reformulated samples as the level of incorporated BPE increased due to the influence of the acidic pH of the extract.

During storage, pH significantly (*p* < 0.05) increased in all samples as a result of the endogenous and exogenous proteolytic, deaminase and deamidase activities resulting in the formation of ammonia and other basic compounds [64]. However, the lowest rate of pH increase during storage was observed in the reformulated samples without BPE incorporation, showing a lower degradative activity.

### 2.7. Texture Analysis of Vienna Sausages

Texture is important in terms of food palatability and can have a strong influence on consumer acceptance. The texture parameters of control and reformulated Vienna sausages at 0, 7 and 14 days of storage at 4 °C are shown in Table 6. Hardness and share force were not significantly (*p* < 0.05) affected by the replacement of pork backfat with the control emulsion gel, while gumminess and chewiness slightly decreased.

The incorporation of BPE resulted in a progressive and significant (*p* < 0.05) increase in hardness, gumminess and share force as the BPE incorporation percentage increased as compared with the control sausages reformulated without extract (VSEG0), the highest values occurring in VSEG2.0 reformulated sausages. The increase in hardness as a result of BPE incorporation could be attributed to the lower emulsion stability of these emulsion gels as compared with the control emulsion gels expressed by the higher total fluid release as previously presented in Table 1, considering that in meat products, water acts as a plasticizer, and a higher water loss results in a hardness increase [65]. In fact, the pH of sausages slightly decreased as the BPE incorporation percentage increased. The lower the pH, the lower the net negative charge on the proteins; consequently, the lower the water holding capacity, with part of the immobilized water becoming free water [66]. The increase in hardness or chewiness parameters is considered to be an unfavorable characteristic from the consumer’s point of view, being associated with reduced meat quality [59].

Reformulation also resulted in an increase in springiness as an indicator of elasticity, probably due to the presence of the hydrocolloid chitosan [53]. The elasticity continued to increase progressively with the incorporation of BPE. Hardness, shear force and gumminess increased during storage in all formulations, in good agreement with the evolution of these texture parameters in the emulsion gels (Table 2). The cohesiveness and resilience of sausages were not significantly affected (*p* < 0.05) during storage, although they decreased in emulsion gels during the storage period.

### 2.8. Cooking Loss and Moisture Retention

The results for cooking loss and moisture retention in control and reformulated Vienna sausages are presented in Table 7. The replacement of pork backfat with the control emulsion gel resulted in an increase in moisture retention accompanied by a reduction in the cooking loss as compared with the traditional product. As shown in previous studies, this improvement in technological properties is due to the good water- and oil-holding capacity of both soy protein isolate and chitosan and to their interaction with the meat proteins, which made it possible to totally replace pork backfat with emulsion gels without negative effects on the texture [25]. The higher fat content of the control sausages as compared with the reformulated ones (Table 3) also contributed to the lower cooking loss of the latter. The highest moisture retention and the lowest cooking loss were found in VSEG0. Moisture retention decreased and cooking loss increased progressively as the level of BPE incorporation increased, mainly as a result of the pH decrease, as previously discussed. As previously reported, cooking loss showed the same trend as total fluid release (Table 1) [53,67]. No significant differences (*p* < 0.05) were found between the cooking loss values of VSEG1.0 and VSC. Berasategi et al. [22] also reported similar weight loss percentages in reformulated Bologna sausages stabilized with *Melissa officinalis* extract as in traditional products.

### 2.9. Lipid Oxidation

**Figure 1:**
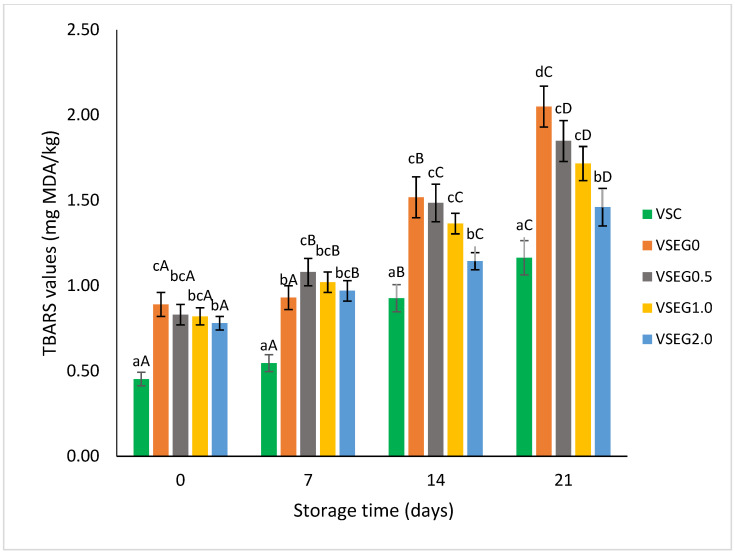
TBARS values (mg MDA/kg) in control and reformulated Vienna sausages at 0, 7, 14 and 21 days of storage at 4 °C. Different lowercase letters indicate significant differences between sausage formulations (*p* < 0.05) for the same storage period while different uppercase letters are indicative of significant differences between sampling times for the same sausage formulation (*p* < 0.05).

Lipid oxidation is responsible for discoloration, the development of unpleasant flavors and odors and the formation of toxic compounds in meat products, thus limiting their shelf-life [68]. TBARS values were monitored during the shelf life in Vienna sausages, and the results are presented in Figure 1. After processing, the TBARS values were higher in reformulated Vienna sausages as compared with the traditional product made with pork backfat. These results are probably due to the higher oxidation degree of the oils incorporated into the emulsion gels replacing the pork backfat. Moreover, the important increase in the content of polyunsaturated fatty acids as a result of reformulation is expected to lead to an augmented susceptibility to oxidation in the meat product [6,69], the incorporation of healthy oils into meat products being known to be difficult due to their high susceptibility to lipid oxidation [70].

At day 0, no significant (*p* < 0.05) differences were found between TBARS values of the control reformulated sausages and those incorporating BPE. Lipid oxidation increased during refrigerated storage both in control and reformulated sausages, but the growth rate of TBARS values decreased progressively during storage as the level of BPE incorporation increased. After processing, all formulations showed TBARS values within acceptable limits, but after 14 days of storage, all reformulated sausages recorded TBARS values above the threshold of 1.0 mg MDA/kg, which is proposed as the reference value for rancidity development in meat products [69]. After 21 days of refrigerated storage, the lowest TBARS values in reformulated sausages were found in VSEG2.0 (1.46 mg MGA/kg), which is significantly lower than in control reformulated Vienna sausages (VSEG0). However, VSEG2.0 exhibited significantly (*p* < 0.05) greater lipid oxidation levels than the control samples made with pork backfat (VSC) throughout the storage period. Differences in TBARS levels between VSEG0 and VSEG2.0 samples were significant (*p* < 0.05) after day 14. These data demonstrate that BPE contributed to the protection of the reformulated sausages against lipid oxidation. Blejan et al. [48] reported that the blackcurrant pomace extracts contain high levels of phenolic compounds, including anthocyanins, that are able to stabilize free radicals by donating hydrogen atoms, and to reduce the rate of oxidation.

Other authors also evaluated the oxidation of reformulated meat products stabilized with plant extracts, and found positive results [12,71]. Berasategi et al. [22] stabilized reduced-fat Bologna sausages enriched in ALA and DHA with *Melissa officinalis* extract, de Oliveira et al. [4] used raspberry extract addition as a strategy to improve the oxidative stability of pork burgers enriched with omega-3 fatty acids, while Noorolahi et al. [72] incorporated pistachio green hull extract into omega-3-rich kappa-carrageenan oleogel used to reformulate dry fermented sausages.

### 2.10. Sensory Analysis

**Figure 2:**
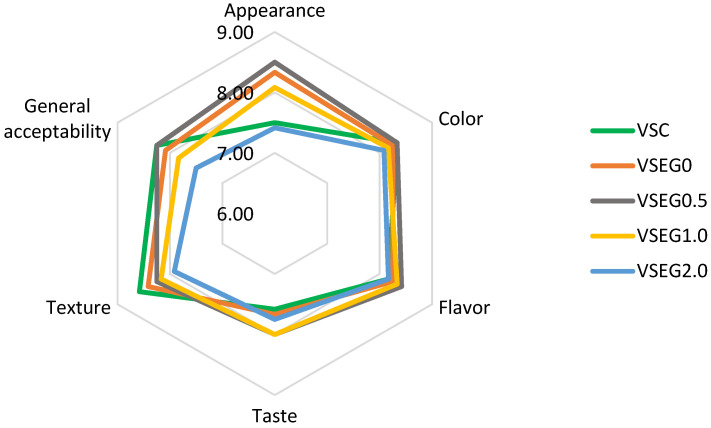
Sensory evaluation of control and reformulated Vienna sausages.

Sensory properties represent one of the limiting factors of reformulation strategies due to the sensorial contribution of the animal fat in meat products. Figure 2 shows the results of the sensory evaluation of control and reformulated Vienna sausages after processing (day 0). Statistical differences are presented in Table S1, available in the “Supplementary Materials” section. The appearance was mainly assessed based on the external color of the sausages. The control samples, made with pork backfat, had a pale pink color, while the reformulated samples had a brown color, becoming darker as the BPE incorporation level increased. VSC and VSEG2.0 received the lowest scores for appearance, the former due to the exceedingly pale color and the other due to the excessively brown color. Some panelists mentioned that they prefer the brown color because they associate it with more intense smoking. VSEG0.5 received the highest scores for appearance, followed by VSEG0. The color assessment concerned the internal color of the Vienna sausages. The highest scores were received by VSC and VSEG0.5, but there were no significant differences (*p* < 0.05) between the formulations regarding the color scores.

The panelists awarded the highest scores for taste to the VSEG0.5 and VSEG1.0 formulations; however, the taste was not significantly affected by the formulation either. De Oliveira et al. [4] also reported that the enrichment of emulsions based on linseed oil and pea protein with raspberry extract did not cause significant changes in the sensory descriptors of reformulated burgers. In terms of texture, the control samples (both VSC and VSEG0) were the best rated, while VSEG2.0 obtained the lowest score, so the incorporation of the extract negatively influenced the texture in a way that was perceived by the panelists. These results are in agreement with the results on the texture parameters, showing the progressive increase in hardness, shear force and gumminess of the sausages as the incorporation level of BPE increased (Figure 2). Regarding the general acceptability, VSEG0.5 received the highest level, while no significant differences (*p* < 0.05) were found among formulations, except for VSEG2.0, which was the lowest-rated.

## 3. Conclusions

In this study, BPE was incorporated at 2.5%, 5% and 10% levels into an emulsion gel based on high-oleic sunflower and flaxseed oils to be used as a pork backfat replacer in Vienna sausages. The reformulation improved the nutritional quality and fatty acid profile of Vienna sausages by decreasing the fat content, the SFA content and, most of all, the n-6/n-3 ratio by more than 7 times, while the incorporation of BPE did not have a major impact on the fatty acid profile or nutritional indices. The replacement of pork backfat with the control emulsion gel improved the technological properties as compared with the traditional product; however, moisture retention decreased and cooking loss increased progressively as the level of BPE incorporation increased, as a result of the reduction in the emulsion gel’s stability. The incorporation of BPE improved color but affected texture by increasing the hardness, gumminess, and share force of sausages as compared with the control reformulated sausages—changes that were negatively perceived by the panelists during the sensory evaluation. BPE increased the oxidative stability during storage; however, the oxidation level was higher in the reformulated sausages as compared with those made with pork backfat over 21 days storage. The incorporation of BPE up to a level of 5% in the emulsion gels did not significantly affect the sensory attributes or general acceptability of the reformulated sausages.

## 4. Materials and Methods

### 4.1. Materials

Blackcurrants (*Ribes nigrum* L.) were harvested from the wild flora of Valcea county (South-West Oltenia Region, Romania) and industrially processed into juice at a commercial juice manufacturer in Vaideeni (Vâlcea county, Romania). Two batches of fresh pomace of 5 kg each, consisting of peels, seeds, and residual pulp, were collected and frozen (at −18 °C) in sealed polyethylene sacks until further use. When needed, the frozen pomaces were thawed in the air at room temperature and dried at 57 °C in a convective laboratory dryer (Deca +SS Design, Profimatic, Cluj-Napoca, Romania). The dried pomaces were ground in a household electric grinder, sieved through a 0.5 mm sieve, and preserved at 20 °C in the dark until use.

Frozen trimmed lean pork meat and pork backfat from a local slaughterhouse were used for Vienna sausage preparation after thawing for 18 h at 2 ± 2 °C. Other ingredients and additives included in the Vienna sausage recipe were nitric salt and potato starch from Daz Activ (Bucharest, Romania), food coloring Propicolor, additives, and a spice mixture CW Super for Vienna sausages from Helmut Grün (Bucharest, Romania) containing phosphates (E 451), dextrose, ascorbic acid (E 300), glucono-delta-lactone (E 575), citric acid (E 330), nutmeg, ginger, sweet paprika and coriander.

For the emulsion gel formulation, soy protein isolate (SPI) (Supro Ex 37, protein content 91.8%) was provided by Solae Belgium N.V. (Ieper, Belgium), while microbial transglutaminase Activa WM, with a standard enzyme activity of 100 U/g, was purchased from Ajinomoto Europe Sales GmbH (Hamburg, Germany) and chitosan was provided by BiOrigins (Fordingbridge, UK). The linseed oil, containing 8% SFA, 22% MUFA and 70% PUFA, according to supplier information, was provided by Herbavit (Oradea, Romania), while the high oleic sunflower oil Vita D’or was purchased from Lidl Discount S.R.L. (Craiova, Romania) and had the following nutritional specifications regarding lipid composition: SFA, 6.9%; MUFA, 70.5%; and PUFA, 14.4%.

### 4.2. Chemicals and Reagents

DPPH (2,2-diphenyl−1-picrylhydrazyl), 6-hydroxy−2,5,7,8-tetramethylchroman-2-carboxylic acid (Trolox), gallic acid, sodium acetate, Folin–Ciocalteu’s phenol reagent, trichloroacetic acid (>99%), thiobarbituric acid (>98%) and malondialdehyde (>96%) were purchased from Sigma-Aldrich (Steinheim, Germany), while anhydrous sodium carbonate, hydrochloric acid, potassium chloride and methanol were provided by Merck (Darmstadt, Germany).

### 4.3. Preparation and Analysis of Blackcurrant Pomace Extract

The blackcurrant pomace extract, which was further used as an ingredient in the emulsion gel formulation, was prepared as follows: 50 g of dry blackcurrant pomace powder was weighed and added to 500 mL of water preheated at 80 °C. The mixture was subjected to sonication for 30 min and filtered through Whatman No.1 filter paper. The extraction of blackcurrant pomace powder was performed in triplicate. The blackcurrant pomace powder and the aqueous extracts were subsequently evaluated for total anthocyanin content, total phenolic content, and 2,2-diphenyl-1-picrylhydrazyl (DPPH) radical-scavenging activity.

The total anthocyanin contents (TACs) of the extracts were determined according to the pH differential method proposed by Lee et al. [73], and the results have been expressed in milligrams of cyanidin-3-O-glucoside equivalents per liter of extract (mg CGE/L). The total phenolic content (TPC) was determined spectrophotometrically following the Folin–Ciocalteu colorimetric method as described by Singleton et al. [74] and expressed in milligrams of Gallic acid equivalents per liter of extract (mg GAE/L), while the DPPH radical-scavenging activity (RSA) of the extracts was evaluated using the spectrophotometric method previously described by Oliveira et al. [75] and expressed in millimoles of Trolox per liter of extract (mmol Trolox/L).

### 4.4. Preparation of Emulsion Gels

Four emulsion gel formulations were manufactured to be used in Vienna sausages as pork backfat replacers: EG0, EG2.5, EG5.0, and EG10.0. These were produced by replacing 0%, 2.5%, 5.0% and 10.0% of the water used in the gel formulation with blackcurrant pomace extract, respectively. Since the emulsion gel represents 20% of the composition (meat + fat), the levels of blackcurrant pomace extract in the composition of Vienna sausage formulations VSEG0, VSEG0.5, VSEG1.0, and VSEG2.0 were 0, 0.5, 1.0, and 2.0%, respectively.

The emulsion gels were prepared according to a slightly modified method described by Pintado and Cofrades [16], which was successfully used by our research group in previous studies [21,76]. Soy protein isolate (8%) was dispersed by stirring in a planetary mixer (Rohnson R586, 700W, Praha, Czech Republic) until solubilization in the aqueous phase (43% water or mixture of water and blackcurrant pomace extract) together with microbial transglutaminase (1%) at 20 °C for 2 min. Chitosan (3%) was added as a cold gelling agent and the composite was further homogenized until complete mixing (3 min). The final blend was emulsified for another 3 min under stirring with continuous inclusion of the oil mixture (45%) consisting of 75% high-oleic sunflower oil and 25% flaxseed oil. The resulting emulsion gels were then placed into plastic containers and stored under refrigeration (4 °C) for 20 h until Vienna sausages’ preparation. The CIELab color parameters, pH, thermal stability and textural properties of the emulsion gels were determined immediately after they had been obtained, as well as after 7 and 14 days of cold storage (4 °C).

### 4.5. Formulation and Processing of Vienna Sausages

Five different formulations of Vienna sausages were manufactured in a meat processing plant (Casa Corina S.R.L., Craiova, Romania). Two batches of 10 kg were prepared for each formulation on different days. Control Vienna sausages (VSC) were prepared using fresh lean minced pork (8 kg), minced pork backfat (2 kg), water (2.63 kg), starch (0.40 kg), nitric salt (0.21 kg), additives, the spice mixture CW Super for Vienna sausages (0.11 kg), and the food coloring Propicolor (0.01 kg). Briefly, the manufacturing process started in a cutter and involved the following steps: (1) fine chopping of meat, (2) adding salt, (3) adding ice/water, (4) adding nitric salt, (5) adding pork backfat/emulsion gel, and (6) adding spices mixture CW Super, additives and food coloring. The meat batter was stuffed into edible bovine collagen casings (Cutisin, Devro s.r.o., Jilemnice, Czech Republic; 20 mm in diameter and 140 in length) using an HP-25 vacuum filler (Vemag Maschinenbau, Verden, Germany). The thermal treatment of sausages was performed in an industrial smoking chamber Fessmann Turbomat 1800 RT (Fessmann, Winnenden, Germany) and consisted of three steps: cooking at 60 °C for 30 min, followed by cooking at 75 °C for 25 min and finally smoking at 60 °C during 6 min. Then, the Vienna sausages were showered with cold water for 10 min and stored under refrigeration (4 °C) for 21 days.

The sausages of the formulations VSEG0, VSEG0.5, VSEG1.0, and VSEG2.0 were manufactured following the same recipe and technology as VSC, except that pork backfat was replaced with EG0, EG2.5, EG5.0, and EG10.0 emulsion gels, respectively.

### 4.6. Emulsion Stability, Color and pH

The emulsion stability, pH, and color of the emulsion gels were assessed the day after processing and after 7 and 14 days of cold storage (4 °C).

Emulsion gel stability was determined in triplicate, based on the procedure described by Jiménez-Colmenero et al. [18] with minor modifications. Emulsion samples (about 25 g) were weighed and stuffed into Falcon tubes, which were sealed and heated in a thermostatic water bath (Labbox, Barcelona, Spain) at 70 °C for 30 min. Then, the tubes were centrifuged at 2500× *g* in a Hermle Z300 centrifuge (Hermle Labortechnik, Wehingen, Germany) for 15 min, opened, and left standing upside down for 50 min to drain out the separated exudate (fat and water). The remaining emulsion was weighed and the total fluid release (TFR) was determined as weight loss and expressed as a percent of the initial sample weight.

The pH was determined by using a Hanna pH meter (model HI255, Hanna Instruments, Padova, Italy). The analyses were performed at room temperature on the emulsion gel water homogenates (1:10, *w*/*v*). Three determinations were performed for each sample.

The colors of the emulsion gels were determined using a PCECSM1 colorimeter (PCE Instruments, Southampton, UK) with spectral reflectance operating in the CIEL*a*b* system. L*, a*, and b* parameters were recorded to indicate lightness, redness, and yellowness, respectively. Six readings were performed for each sample at different randomly selected points of fresh-cut sections of the emulsion gels to obtain a representative mean value.

### 4.7. Proximate Composition and Energy Value

Proximate composition analyses were performed on Vienna sausage samples the day after processing, with three replicates per analysis. The quantification of moisture, fat, protein, and ash contents was performed following the methods described by the Association of Official Analytical Chemists [77], while carbohydrate content was estimated by difference. The moisture content was determined by the moisture loss of the sample maintained at 105 °C in a Memmert ULM500 oven (Uden, The Netherlands) (AOAC Method 950.46), the fat content was determined by Soxhlet extraction in a Soxhlet automatic extraction system (SER 148/3, Velp Scientific, Usmate, Italy), and ash content was assessed by sample incineration at 550 °C in a Caloris CL 1206 oven (Caloris Group S.A., Bucharest, Romania) (AOAC Method 940.26). The protein content was quantified after multiplying by 6.25 the nitrogen content determined by Kjeldahl digestion in an automated nitrogen analyzer (UDK 149 Velp Scientific, Milan, Italy) (AOAC Method 920.152). Energy values were calculated based on 4 kcal/g for protein and carbohydrate contents and 9 kcal/g for fat content.

### 4.8. Fatty Acid Profile and Nutritional Indices

The fatty acid composition was determined in triplicate by gas chromatography with flame ionization detection in a Perkin–Elmer gas chromatograph (model Clarus 500, Shelton, MA, USA) after the conversion of fatty acids to their methyl esters (FAMEs) by 4 h of transesterification in methanol containing 3% concentrated sulfuric acid at 80 °C.

FAMEs were separated on a DB-23 GC capillary column (60 m × 0.25 mm id × 0.25 µm film thickness) from Agilent J&W GC Columns (Santa Clara, CA, USA) heated from 180 °C to 220 °C with a ramp rate of 5 °C/min. Hydrogen was the carrier gas appllied at a flow rate of 35 cm/s and a split ratio of 1:100. FAMEs were detected by retention time and identified by comparison with individual standards provided by Sigma-Aldrich Chemical Co. (St. Louis, MO, USA). The fatty acid contents were calculated based on peak areas and were expressed in grams of fatty acids per 100 g of total fatty acids.

The fatty acids content was used to calculate the sum of saturated (SFA), monounsaturated (MUFA), polyunsaturated (PUFA), n-3 polyunsaturated (n-3 PUFA), and n-6 polyunsaturated (n-6 PUFA) fatty acids, as well as the PUFA/SFA, MUFA/SFA, and n-6/n-3 ratios. The atherogenic (AI) and thrombogenic (TI) indexes were determined according to Ulbricht and Southgate [78], and the ratio of hypocholesterolemic and hypercholesterolemic fatty acids (h/H) was calculated according to Santos-Silva et al. [79].

AI = (C12:0 + 4 × C14:0 + C16:0)/(MUFA + PUFA)

TI = (C14:0 + C16:0 + C18:0)/(0.5 × MUFA + 0.5 × PUFA n-6 + 3 × PUFA n-3 + PUFA n-3/PUFA n-6)

h/H = (C18:1 n-9 + C18:2 n-6 + C20:4 n-6 + C18:3 n-3 + C20:5 n-3 + C22:5 n-3 +C22:n-3)/(C14:0 + C16:0)

### 4.9. Color and pH of Vienna Sausages

Vienna sausages were evaluated for internal color using a PCECSM1 colorimeter (PCE Instruments, Southampton, UK) with spectral reflectance, operating in the CIEL*a*b* system, calibrated against a standard white plate. L*, a*, and b* color coordinates indicating lightness, redness, and yellowness, respectively, were determined the day after processing and after 7, 14, and 21 days of refrigerated storage (4 °C). The determinations were performed at three different points of six fresh-cut sections of Vienna sausage samples, and the average of each sample was used in the statistical analysis. The pH of sausages was measured with the same frequency as the color by using a Hanna pH meter (model HI255, Hanna Instruments, Padova, Italy). The pH determinations were performed at room temperature on homogenates made from finely ground sausage samples and water in a ratio of 1:10 (*w*/*v*).

### 4.10. Cooking Loss and Moisture Retention

The Vienna sausage batches were weighted before thermal treatment. After thermal treatment, showering and chilling overnight at 2 °C, the Vienna sausage batches were weighed again and the cooking loss was calculated as follows [80]:

Cooking loss (%) = [(weight_raw_ − weight_cooked_)/weight_raw_] × 100

The moisture retention, representing the amount of moisture retained in the processed product per 100 g of sample, was calculated according to the following equation [81]:

Moisture retention (%) = [(100 − cooking loss (%)) × moisture_cooked_]/100.

### 4.11. Analysis of the Textural Parameters

To evaluate the textural characteristics of Vienna sausage samples, a double compression test and a cutting test were performed using a TVT-6700 texturometer (Perten Instruments, Hägersten, Sweden) equipped with a 10 kg load cell. In the compression test, the samples were subjected to two compression cycles, up to 50% of their initial height, using a cylindrical probe of 15 mm height and 20 mm diameter. The starting distance from the sample was 5 mm. Force–time curves were recorded and the following parameters were determined: hardness (N), adhesiveness (J), resilience (dimensionless), springiness (dimensionless), cohesiveness (dimensionless), gumminess (N), and chewiness (N). For each sample, the determinations were performed in triplicate. The cutting test allows the evaluation of the force required to cut the sample (shear force). For the cutting test, the texturometer was equipped with a knife blade 117 mm high and a probe holder. The determinations were carried out in the center of each sample at a test speed of 1.5 mm/s, a starting distance from the sample of 5 mm, and a trigger force of 40 g. The texture profiles of Vienna sausages were determined the day after processing and after 7, 14, and 21 days of refrigerated storage (4 °C).

To evaluate the textural characteristics of emulsion gels, a double compression was applied to samples of 8 g weight (45 mm height and 25 mm diameter) up to 5% of their initial height, at a test speed of 5.0 mm/s, a retraction speed of 5 mm/s, and a trigger force of 10 g. The pause time between compressions was 10 s. The analyses were carried out the day after processing and after 7 and 14 days of refrigerated storage (4 °C). All determinations were made in triplicate.

### 4.12. Lipid Oxidation

To evaluate the lipid oxidation of Vienna sausages, the Thiobarbituric Acid Reactive Substances (TBARS) assay was carried out according to the method described by Witte et al. [82]. Briefly, 5 g of ground sausage and 12.5 mL of 20% trichloroacetic were vortexed for 30 s, then diluted up to 25 mL with cold distilled water. The mixture was centrifuged at 2500 g for 10 min. From the supernatant, 5 mL was collected and mixed with 5 mL of 0.02 M 2-thiobarbituric acid. Finally, the mixture was heated at 100 °C for 35 min, and after cooling in an ice bath to room temperature, the absorbance was measured at 532 nm with a Varian Cary 50 UV spectrophotometer (Varian Co., Palo Alto, CA, USA). A calibration curve was plotted with 1,1,3,3-tetraethoxypropane using the same procedure, and the results have been expressed as mg of malondialdehyde (MDA) equivalents per kg of sample. The analyses were performed the day after processing and after 7, 14, and 21 days of refrigerated storage (4 °C).

### 4.13. Sensory Evaluation

The sensory analysis of control and reformulated Vienna sausages was carried out on the day after processing by eighteen panelists who were selected from the staff and master students of the Food Science Department of the University of Craiova (Craiova, Romania). Each panelist scored the samples for external appearance, internal color, aroma, flavor, texture, and overall acceptance using a 9-point hedonic scale ranging from 1 indicating “dislike very much” to 9 indicating “like very much”. Water and bread were provided to the panelists for cleaning and rinsing the palate between samples. Coded samples were presented to the panelists in a random order after heating in boiling water for 3 min and cutting into cylinders 2.5 cm in length. Triplicate evaluations were performed for each sample and average scores were calculated for each sensory attribute.

### 4.14. Statistical Analysis

The experiment (manufacture of emulsion gels and Vienna sausages) was conducted two times on separate days, and data were analyzed using the Statgraphics Centurion XVI software (StatPoint Technologies, Warrenton, VA, USA). One-way analysis of variance (ANOVA) followed by multiple comparisons of means using the Fisher‘s least significant difference (LSD) test at a 95.0% confidence level was applied to find the statistical significance of the effect of emulsion gel formulation on proximate composition, fatty acid profile, cooking loss and sensory attributes of Vienna sausages, while two-way ANOVA followed by the LSD test (*p* < 0.05) was run to investigate the effects of emulsion gel formulation and storage period on pH values, color parameters, textural characteristics and TBARS values of emulsion gels and Vienna sausages.

## Figures and Tables

**Table 1:** Color parameters (L*—lightness, a*—redness and b*—yellowness), pH and total fluid release of emulsion gels at 0, 7 and 14 days of storage at 4 °C *.

Storage Time (Days)	EG0	EG2.5	EG5.0	EG10.0
		L*		
0	71.76 ± 0.71 ^cA^	68.51 ± 0.75 ^bA^	66.71 ± 1.88 ^bB^	60.81 ± 1.65 ^aB^
7	72.88 ± 0.57 ^dA^	68.99 ± 1.20 ^cA^	65.41 ± 1.37 ^bAB^	57.00 ± 1.67 ^aA^
14	71.62 ± 2.77 ^dA^	68.29 ± 2.93 ^cA^	63.59 ± 1.17 ^bA^	55.56 ± 1.74 ^aA^
		a*		
0	3.59 ± 0.19 ^bAB^	2.99 ± 0.16 ^aA^	2.88 ± 0.18 ^aA^	2.88 ± 0.15 ^aA^
7	3.39 ± 0.10 ^cA^	3.08 ± 0.09 ^abA^	3.01 ± 0.18 ^aA^	3.21 ± 0.18 ^bcB^
14	3.69 ± 0.13 ^bB^	3.33 ± 0.11 ^aB^	3.40 ± 0.23 ^aB^	3.51 ± 0.18 ^abC^
		b*		
0	19.89 ± 0.58 ^dA^	17.45 ± 0.20 ^cA^	16.19 ± 0.18 ^bA^	14.69 ± 0.24 ^aA^
7	19.42 ± 0.09 ^dA^	17.68 ± 0.14 ^cA^	15.72 ± 0.26 ^bA^	14.77 ± 0.35 ^aA^
14	19.33 ± 0.69 ^cA^	17.83 ± ±0.32 ^bA^	16.23 ± 0.46 ^aA^	15.78 ± 0.65 ^aA^
Total fluid release (%)				
0	1.31 ± 0.15 ^aA^	3.45 ± 0.21 ^bA^	5.81 ± 0.22 ^cA^	16.77 ± 0.68 ^dA^
7	2.36 ± 0.19 ^aB^	4.85 ± 0.28 ^bB^	7.24 ± 0.26 ^cB^	21.67 ± 0.88 ^dB^
14	3.24 ± 0.22 ^aC^	8.21 ± 0.45 ^bC^	9.42 ± 0.38 ^bC^	24.33 ± 1.13 ^cC^
pH				
0	8.36 ± 0.03 ^dB^	8.16 ± 0.02 ^cA^	7.87 ± 0.03 ^bA^	7.61 ± 0.02 ^aA^
7	8.42 ± 0.03 ^dAB^	8.18 ± 0.03 ^cA^	8.03 ± 0.04 ^bB^	7.82 ± 0.01 ^aB^
14	8.47 ± 0.04 ^cA^	8.21 ± 0.03 ^bA^	8.16 ± 0.02 ^bC^	7.93 ± 0.03 ^aC^

* Different lowercase letters indicate significant differences between emulsion gel formulations (*p* < 0.05) with the same storage periods, while different uppercase letters are indicative of significant differences between sampling times for the same emulsion gel formulation (*p* < 0.05).

**Table 2:** Texture properties of emulsion gels at 0, 7 and 14 days of storage at 4 °C *.

Storage Time (Days)	EG0	EG2.5	EG5.0	EG10.0
		Hardness (N)		
0	24.82 ± 0.31 ^bA^	21.43 ± 0.31 ^aA^	21.69 ± 0.63 ^aA^	22.00 ± 0.75 ^aA^
7	25.53 ± 0.30 ^aAB^	25.09 ± 0.55 ^aB^	25.63 ± 1.87 ^aB^	26.15 ± 0.82 ^aB^
14	26.61 ± 0.97 ^aB^	29.08 ± 0.78 ^bC^	30.27 ± 0.49 ^bcC^	31.61 ± 0.94 ^cC^
		Cohesiveness		
0	1.23 ± 0.01 ^cB^	1.16 ± 0.03 ^bcB^	1.09 ± 0.08 ^abB^	1.06 ± 0.01 ^aB^
7	1.20 ± 0.01 ^dB^	1.14 ± 0.02 ^cB^	1.07 ± 0.01 ^bB^	1.04 ± 0.03 ^aB^
14	1.02 ± 0.04 ^bA^	1.05 ± 0.04 ^bA^	0.92 ± 0.02 ^aA^	0.90 ± 0.08 ^aA^
		Springiness		
0	1.00 ± 0.01 ^aB^	1.02 ± 0.02 ^aA^	1.06 ± 0.03 ^aB^	1.08 ± 0.09 ^aA^
7	0.97 ± 0.03 ^aAB^	1.10 ± 0.10 ^bA^	1.08 ± 0.05 ^bB^	1.09 ± 0.03 ^bA^
14	0.94 ± 0.03 ^aA^	0.98 ± 0.09 ^aA^	0.98 ± 0.01 ^aA^	1.02 ± 0.09 ^aA^
Adhesiveness (J)				
0	−33.20 ± 0.90 ^dA^	−50.71 ± 1.66 ^cA^	−64.96 ± 0.47 ^bA^	−96.74 ± 1.14 ^aA^
7	−31.78 ± 0.65 ^dAB^	−47.82 ± 0.78 ^cB^	−62.97 ± 0.71 ^bB^	−75.65 ± 0.58 ^aB^
14	−31.03 ± 0.84 ^dB^	−46.20 ± 0.59 ^cB^	−61.03 ± 0.89 ^bC^	−71.39 ± 0.37 ^aC^
Resilience				
0	30.68 ± 0.17 ^dB^	28.92 ± 0.60 ^cC^	27.60 ± 0.88 ^bB^	25.83 ± 0.61 ^aB^
7	30.13 ± 0.56 ^cB^	26.99 ± 0.45 ^bB^	26.69 ± 0.84 ^bB^	24.93 ± 0.58 ^aB^
14	23.67 ± 1.07 ^bA^	25.76 ± 0.67 ^cA^	24.05 ± 0.39 ^bA^	21.24 ± 0.78 ^aA^
		Gumminess (N)		
0	24.82 ± 0.31 ^bA^	23.67 ± 0.85 ^aA^	23.76 ± 0.32 ^abA^	24.02 ± 0.66 ^abA^
7	26.86 ± 0.84 ^aB^	25.82 ± 0.58 ^aB^	26.09 ± 0.55 ^aB^	26.30 ± 0.53 ^aB^
14	27.28 ± 0.64 ^aC^	28.28 ± 0.97 ^abC^	30.08 ± 1.76 ^bC^	32.27 ± 0.49 ^cC^
		Chewiness (N)		
0	24.18 ± 0.76 ^bA^	20.73 ± 0.51 ^aA^	20.96 ± 0.85 ^aA^	20.99 ± 0.23 ^aA^
7	26.41 ± 0.67 ^bcB^	25.58 ± 0.56 ^abB^	24.98 ± 0.41 ^aB^	27.12 ± 0.96 ^aB^
14	27.74 ± 0.50 ^aC^	28.75 ± 0.73 ^bC^	32.41 ± 0.18 ^cC^	36.41 ± 0.39 ^dC^

* Different lowercase letters indicate significant differences between emulsion gel formulations (*p* < 0.05) with the same storage period, while different uppercase letters are indicative of significant differences between sampling times for the same emulsion gel formulation (*p* < 0.05).

**Table 3:** Proximate composition and energy values of control and reformulated Vienna sausages *.

	VSC	VSEG0	VSEG0.5	VSEG1.0	VSEG2.0
Moisture (%)	58.57 ± 1.33 ^a^	61.46 ± 0.97 ^b^	59.95 ± 1.11 ^ab^	59.88 ± 1.48 ^ab^	60.67 ± 0.83 ^ab^
Protein (%)	12.66 ± 0.45 ^a^	12.82 ± 0.61 ^a^	13.21 ± 0.38 ^a^	13.15 ± 0.49 ^a^	13.34 ± 0.55 ^a^
Fat (%)	20.32 ± 0.48 ^c^	17.52 ± 0.52 ^a^	18.99 ± 0.35 ^b^	18.62 ± 0.51 ^b^	18.22 ± 0.67 ^ab^
Ash (%)	2.26 ± 0.16 ^a^	2.46 ± 0.09 ^a^	2.39 ± 0.11 ^a^	2.45 ± 0.14 ^a^	2.42 ± 0.13 ^a^
Energy value (kcal/100 g)	267.32 ± 2.92 ^d^	241.76 ± 1.28 ^a^	255.15 ± 2.69 ^c^	253.58 ± 3.37 ^c^	248.42 ± 1.93 ^bc^
Energy from fat (kcal/100 g)	182.88 ± 4.32 ^c^	157.68 ± 4.68 ^a^	170.91 ± 3.15 ^b^	167.58 ± 4.59 ^b^	163.98 ± 6.03 ^ab^
Fat reduction (%)	-	13.79 ± 0.52 ^d^	6.54 ± 0.49 ^a^	8.37 ± 0.35 ^b^	10.35 ± 1.18 ^c^
Energy value reduction (%)	-	9.56 ± 0.51 ^c^	4.55 ± 0.04 ^a^	5.14 ± 0.22 ^a^	7.06 ± 1.03 ^b^

* Different lowercase letters indicate significant differences between sausage formulations (*p* < 0.05).

**Table 4:** Fatty acid profile (expressed as g/100 g of total fatty acids) and nutritional indices of control and reformulated Vienna sausages *.

Fatty Acids	VSC	VSEG0	VSEG0.5	VSEG1.0	VSEG2.0
Caproic (C6:0)	0.05 ± 0.01 ^a^	0.11 ± 0.01 ^c^	0.09 ± 0.02 ^bc^	0.07 ± 0.01 ^ab^	0.09 ± 0.01 ^bc^
Caprylic (C8:0)	0.18 ± 0.02 ^c^	0.15 ± 0.02 ^bc^	0.12 ± 0.02 ^ab^	0.11 ± 0.01 ^a^	0.10 ± 0.01 ^a^
Capric (C10:0)	0.21 ± 0.02 ^c^	0.11 ± 0.02 ^b^	0.10 ± 0.01 ^ab^	0.11 ± 0.01 ^b^	0.08 ± 0.01 ^a^
Myristic (C14:0)	1.77 ± 0.08 ^b^	0.94 ± 0.06 ^a^	1.03 ± 0.05 ^a^	0.94 ± 0.04 ^a^	0.93 ± 0.05 ^a^
Palmitic (C16:0)	21.87 ± 0.89 ^b^	13.99 ± 0.58 ^a^	14.14 ± 0.56 ^a^	13.83 ± 0.65 ^a^	14.01 ± 0.48 ^a^
Palmitoleic (C16:1n-7)	3.25 ± 0.15 ^b^	1.55 ± 0.08 ^a^	1.57 ± 0.10 ^a^	1.58 ± 0.09 ^a^	1.54 ± 0.12 ^a^
Heptadecanoic (C17:0)	0.21 ± 0.02 ^b^	0.17 ± 0.02 ^a^	0.18 ± 0.02 ^ab^	0.15 ± 0.01 ^a^	0.17 ± 0.02 ^a^
Heptadecenoic (C17:1n-7)	0.28 ± 0.03 ^b^	0.15 ± 0.02 ^a^	0.17 ± 0.02 ^a^	0.15 ± 0.02 ^a^	0.15 ± 0.02 ^a^
Stearic (C18:0)	11.20 ± 0.45 ^b^	7.84 ± 0.34 ^a^	7.85 ± 0.28 ^a^	7.90 ± 0.40 ^a^	7.98 ± 0.35 ^a^
Oleic (C18:1n-9)	43.25 ± 1.20 ^a^	49.33 ± 1.66 ^b^	49.15 ± 0.89 ^b^	49.35 ± 1.23 ^b^	49.51 ± 1.35 ^b^
Linoleic (C18:2n-6)	14.76 ± 0.36 ^a^	14.49 ± 0.22 ^a^	14.23 ± 0.52 ^a^	14.18 ± 0.48 ^a^	14.17 ± 0.54 ^a^
α-Linolenic (C18:3n-3)	0.93 ± 0.04 ^a^	9.33 ± 0.44 ^b^	9.27 ± 0.36 ^b^	9.40 ± 0.38 ^b^	9.39 ± 0.43 ^b^
Octadecatetraenoic (C18:4n-3)	0.52 ± 0.03 ^a^	0.51 ± 0.02 ^a^	0.55 ± 0.03 ^ab^	0.59 ± 0.02 ^b^	0.64 ± 0.03 ^c^
Eicosadienoic (C20:2n-6)	0.51 ± 0.02 ^c^	0.40 ± 0.02 ^a^	0.45 ± 0.03 ^b^	0.45 ± 0.02 ^b^	0.43 ± 0.02 ^ab^
Arachidonic (C20:4n-6)	0.67 ± 0.03 ^b^	0.58 ± 0.03 ^a^	0.57 ± 0.03 ^a^	0.57 ± 0.02 ^a^	0.56 ± 0.02 ^a^
Other fatty acids	0.34 ± 0.02 ^a^	0.35 ± 0.02 ^a^	0.53 ± 0.03 ^b^	0.62 ± 0.03 ^c^	0.38 ± 0.03 ^a^
Nutritional indices					
SFA	35.49 ± 1.49 ^b^	23.31 ± 1.05 ^a^	23.51 ± 0.96 ^a^	23.11 ± 1.13 ^a^	23.36 ± 0.93 ^a^
MUFA	46.78 ± 1.38 ^a^	51.03 ± 1.76 ^b^	50.89 ± 1.01 ^b^	51.08 ± 1.34 ^b^	51.20 ± 1.49 ^b^
PUFA	17.39 ± 0.48 ^a^	25.31 ± 0.73 ^b^	25.07 ± 0.97 ^b^	25.19 ± 0.92 ^b^	25.19 ± 1.04 ^b^
PUFA n-6	15.94 ± 0.41 ^a^	15.47 ± 0.27 ^a^	15.25 ± 0.58 ^a^	15.20 ± 0.52 ^a^	15.16 ± 0.58 ^a^
PUFA n-3	1.45 ± 0.07 ^a^	9.84 ± 0.46 ^b^	9.82 ± 0.39 ^b^	9.99 ± 0.40 ^b^	10.03 ± 0.46 ^b^
n-6/n-3	10.99	1.57	1.55	1.52	1.51
AI	0.45	0.23	0.24	0.23	0.23
TI	0.97	0.36	0.36	0.36	0.36
h/H	2.52	4.94	4.83	4.98	4.92

* Different lowercase letters indicate significant differences between sausage formulations (*p* < 0.05).

**Table 5:** Color parameters (L*—lightness, a*—redness and b*—yellowness) and pH of control and reformulated Vienna sausages at 0, 7, 14 and 21 days of storage at 4 °C *.

Storage Time (Days)	VSC	VSEG0	VSEG0.5	VSEG1.0	VSEG2.0
L*					
0	67.28 ± 1.09 ^aC^	70.12 ± 0.70 ^cC^	69.94 ± 0.62 ^cB^	68.82 ± 0.56 ^bB^	68.32 ± 0.45 ^bB^
7	67.22 ± 0.69 ^aBC^	68.88 ± 0.75 ^bBC^	69.33 ± 0.55 ^bB^	67.65 ± 0.75 ^aA^	67.86 ± 0.72 ^aB^
14	65.74 ± 0.66 ^aAB^	67.41 ± 0.81 ^bAB^	67.83 ± 0.89 ^bA^	67.37 ± 1.27 ^bA^	66.60 ± 1.05 ^abA^
21	64.29 ± 1.75 ^aA^	66.14 ± 1.86 ^bA^	67.08 ± 1.08 ^bA^	66.67 ± 0.39 ^bA^	65.82 ± 0.87 ^abA^
a*					
0	15.24 ± 0.45 ^dAB^	13.75 ± 0.55 ^aA^	14.07 ± 0.22 ^abAB^	14.55 ± 0.16 ^cAB^	14.30 ± 0.11 ^bcB^
7	14.73 ± 0.11 ^bcA^	14.06 ± 0.25 ^aAB^	13.94 ± 0.07 ^aA^	14.93 ± 0.21 ^cC^	14.52 ± 0.25 ^bB^
14	14.98 ± 0.32 ^bA^	14.38 ± 0.35 ^aB^	14.36 ± 0.31 ^aBC^	14.71 ± 0.24 ^abBC^	14.46 ± 0.30 ^aB^
21	15.59 ± 0.57 ^cB^	13.87 ± 0.62 ^abAB^	14.43 ± 0.26 ^bC^	14.29 ± 0.23 ^abA^	13.80 ± 0.41 ^aA^
b*					
0	12.28 ± 0.23 ^aA^	12.58 ± 0.26 ^abA^	12.39 ± 0.25 ^abA^	12.65 ± 0.19 ^bA^	12.45 ± 0.26 ^abA^
7	12.10 ± 0.05 ^aA^	12.42 ± 0.08 ^bA^	12.34 ± 0.14 ^abA^	12.70 ± 0.31 ^cA^	12.67 ± 0.23 ^cA^
14	12.75 ± 0.14 ^aB^	13.03 ± 0.19 ^bcB^	12.91 ± 0.25 ^abB^	13.09 ± 0.10 ^bcB^	13.13 ± 0.06 ^cB^
21	12.77 ± 0.21 ^aB^	13.07 ± 0.19 ^bB^	13.26 ± 0.35 ^bC^	13.28 ± 0.15 ^bB^	13.77 ± 0.05 ^cC^
pH					
0	6.24 ± 0.03 ^bcA^	6.47 ± 0.04 ^dA^	6.30 ± 0.04 ^cA^	6.19 ± 0.03 ^bA^	6.10 ± 0.04 ^aA^
7	6.40 ± 0.05 ^bB^	6.54 ± 0.03 ^cB^	6.41 ± 0.05 ^bB^	6.29 ± 0.04 ^aB^	6.22 ± 0.03 ^aB^
14	6.48 ± 0.04 ^bB^	6.56 ± 0.03 ^cB^	6.59 ± 0.06 ^cC^	6.57 ± 0.03 ^cC^	6.33 ± 0.04 ^aC^
21	6.94 ± 0.04 ^bcC^	6.78 ± 0.04 ^aC^	6.80 ± 0.05 ^aD^	6.91 ± 0.06 ^bD^	7.01 ± 0.05 ^cD^

* Different lowercase letters indicate significant differences between sausage formulations (*p* < 0.05) with the same storage period while different uppercase letters are indicative of significant differences between sampling times for the same sausage formulation (*p* < 0.05).

**Table 6:** Texture parameters of control and reformulated Vienna sausages at 0, 7 and 14 days of storage at 4 °C *.

Storage Time (Days)	VSC	VSEG0	VSEG0.5	VSEG1.0	VSEG2.0
Hardness (N)					
0	32.47 ± 0.63 ^bA^	29.91 ± 1.15 ^aA^	39.97 ± 1.83 ^cA^	39.25 ± 0.22 ^cA^	41.18 ± 1.44 ^cA^
7	38.33 ± 1.01 ^abB^	37.43 ± 0.61 ^aB^	42.48 ± 1.42 ^cAB^	39.43 ± 1.25 ^bA^	41.79 ± 0.58 ^cA^
14	38.71 ± 1.97 ^aB^	37.76 ± 1.95 ^aB^	43.04 ± 1.01 ^bB^	41.90 ± 1.14 ^bB^	46.83 ± 1.90 ^cB^
Cohesiveness					
0	0.84 ± 0.02 ^cB^	0.79 ± 0.04 ^abA^	0.77 ± 0.02 ^abA^	0.82 ± 0.02 ^bcA^	0.77 ± 0.01 ^aA^
7	0.79 ± 0.05 ^aB^	0.79 ± 0.01 ^aA^	0.79 ± 0.01 ^aA^	0.83 ± 0.04 ^aA^	0.80 ± 0.01 ^aA^
14	0.63 ± 0.09 ^aA^	0.80 ± 0.02 ^bA^	0.79 ± 0.02 ^bA^	0.80 ± 0.02 ^bA^	0.78 ± 0.05 ^bA^
Springiness					
0	0.51 ± 0.10 ^aA^	0.65 ± 0.07 ^bA^	0.87 ± 0.09 ^cA^	1.18 ± 0.06 ^dA^	1.21 ± 0.05 ^dA^
7	1.01 ± 0.09 ^aB^	1.11 ± 0.06 ^aB^	1.14 ± 0.06 ^aB^	1.29 ± 0.10 ^bAB^	1.35 ± 0.04 ^bA^
14	1.23 ± 0.32 ^abB^	1.08 ± 0.10 ^aB^	1.24 ± 0.03 ^abB^	1.38 ± 0.09 ^bcB^	1.56 ± 0.10 ^cB^
Adhesiveness (J)					
0	−27.45 ± 4.47 ^aC^	−13.54 ± 1.05 ^bC^	−13.26 ± 0.51 ^bcC^	−9.44 ± 1.22 ^cC^	−14.78 ± 0.68 ^bC^
7	−53.88 ± 3.14 ^aA^	−34.23 ± 1.69 ^cA^	−36.82 ± 1.98 ^cA^	−40.83 ± 1.87 ^bA^	−25.69 ± 1.65 ^dA^
14	−38.87 ± 1.46 ^aB^	−21.97 ± 0.69 ^cB^	−32.25 ± 1.40 ^cB^	−22.50 ± 1.15 ^bB^	−22.13 ± 1.34 ^cB^
Resilience					
0	0.25 ± 0.02 ^abB^	0.27 ± 0.02 ^bA^	0.24 ± 0.01 ^aA^	0.26 ± 0.01 ^abA^	0.25 ± 0.01 ^abA^
7	0.22 ± 0.01 ^aB^	0.24 ± 0.01 ^abA^	0.26 ± 0.01 ^bA^	0.25 ± 0.02 ^abA^	0.25 ± 0.00 ^bA^
14	0.20 ± 0.01 ^aA^	0.25 ± 0.01 ^bA^	0.25 ± 0.01 ^bA^	0.25 ± 0.01 ^bA^	0.26 ± 0.00 ^bA^
Gumminess (N)					
0	27.33 ± 1.24 ^bA^	24.16 ± 0.62 ^aA^	27.33 ± 1.16 ^bA^	31.41 ± 0.98 ^cA^	32.55 ± 0.41 ^cA^
7	31.67 ± 0.62 ^bB^	27.89 ± 1.37 ^aB^	27.46 ± 2.37 ^aA^	32.80 ± 0.17 ^bAB^	33.43 ± 0.90 ^bAB^
14	33.10 ± 1.42 ^bB^	28.07 ± 1.30 ^aB^	32.31 ± 1.08 ^bB^	33.84 ± 1.41 ^bB^	34.24 ± 0.76 ^bB^
Chewiness (N)					
0	27.35 ± 1.23 ^bA^	24.16 ± 1.62 ^aA^	32.42 ± 0.89 ^cA^	26.34 ± 2.04 ^abA^	33.87 ± 1.76 ^cA^
7	31.69 ± 2.62 ^bcB^	27.58 ± 2.81 ^abAB^	34.14 ± 2.29 ^cA^	26.46 ± 1.33 ^aA^	32.44 ± 2.65 ^cA^
14	26.25 ± 1.86 ^aA^	30.72 ± 1.81 ^bB^	33.84 ± 1.41 ^bcA^	34.31 ± 1.53 ^cB^	34.23 ± 2.42 ^cA^
Shear force (N)					
0	13.20 ± 0.93 ^aA^	12.09 ± 0.78 ^aA^	13.67 ± 1.02 ^aA^	16.71 ± 1.23 ^bA^	20.11 ± 0.52 ^cA^
7	13.75 ± 0.91 ^aA^	12.54 ± 1.10 ^aA^	16.19 ± 1.52 ^bA^	17.76 ± 0.91 ^bA^	24.98 ± 0.38 ^cB^
14	17.70 ± 0.79 ^bB^	14.41 ± 1.62 ^aA^	22.78 ± 1.20 ^cB^	28.02 ± 1.34 ^dB^	34.58 ± 1.13 ^eC^

* Different lowercase letters indicate significant differences between sausage formulations (*p* < 0.05) for the same storage period, while different uppercase letters are indicative of significant differences between sampling times for the same sausage formulation (*p* < 0.05).

**Table 7:** Cooking loss and moisture retention of control and reformulated Vienna sausages *.

	VSC	VSEG0	VSEG0.5	VSEG1.0	VSEG2.0
Cooking loss (%)	11.28 ± 0.44 ^b^	8.21 ± 0.36 ^a^	9.02 ± 0.41 ^a^	10.69 ± 0.54 ^b^	13.74 ± 0.59 ^c^
Moisture retention (%)	51.96 ± 0.92 ^a^	56.41 ± 0.67 ^d^	54.54 ± 0.76 ^c^	53.47 ± 1.00 ^bc^	52.33 ± 0.36 ^ab^

* Different lowercase letters indicate significant differences between sausage formulations (*p* < 0.05).

## Data Availability

Data are contained within the article.

## Supplementary Materials

The following supporting information can be downloaded at: https://www.mdpi.com/2310-2861/10/8/534/s1, Table S1: Sensory attributes and overall acceptability scores of control and reformulated Vienna sausages.
