# Supplementary material for: Blackcurrant Pomace Extract as a Natural Antioxidant in Vienna Sausages Reformulated by Replacement of Pork Backfat with Emulsion Gels Based on High Oleic Sunflower and Flaxseed Oils
Source: Gels. 2024 Aug 13;10(8):534. doi: 10.3390/gels10080534 (PMC11487386; doi:10.3390/gels10080534)
Supplement: Supplementary file 1 [file gels-10-00534-s001.zip › gels-3132842-supplementary.pdf]

Table S1. Sensory attributes and overall acceptability scores of control and reformulated Vienna sausages*

	VSC	VSEG0	VSEG0.5	VSEG1.0	VSEG2.0
Appearance	7.50±0.52^{a}	8.33±0.65^{b}	8.50±0.67^{b}	8.08±0.67^{b}	7.42±0.67^{a}
Color	8.33±0.49^{a}	8.25±0.62^{a}	8.33±0.49^{a}	8.17±0.83^{a}	8.08±0.67^{a}
Flavor	8.17±0.72^{a}	8.25±0.62^{a}	8.42±0.79^{a}	8.33±0.65^{a}	8.17±0.72^{a}
Taste	7.58±1.08^{a}	7.67±1.50^{a}	8.00±1.13^{a}	8.00±1.95^{a}	7.75±1.96^{a}
Texture	8.58±0.51^{b}	8.42±0.51^{b}	8.25±0.62^{ab}	8.17±0.72^{ab}	7.92±0.51^{a}
General acceptability	8.25±0.62^{b}	8.08±0.51^{b}	8.25±0.79^{b}	7.83±0.58^{ab}	7.50±0.52^{a}

* Different lowercase letters indicate significant differences between formulations ($p < 0.05$);
